# Implementation and real-world effectiveness of a weighted blanket intervention and standard melatonin treatment for children with ADHD and sleep problems: protocol for a pragmatic longitudinal cohort study

**DOI:** 10.1186/s12888-026-08538-4

**Published:** 2026-08-25

**Authors:** Ingrid Larsson, Petra Svedberg, Håkan Jarbin, Jens Nygren, Annelie Lindholm, Katarina Aili, Julia S. Malmborg

**Affiliations:** 1 School of Health and Welfare, Halmstad University, Box 823, Halmstad, SE-301 18 Sweden; 2 Faculty of Medicine, Lund University, Lund, Sweden; 3 Child and Adolescent Psychiatry, Region Halland, Halmstad, Sweden

**Keywords:** Attention-deficit/hyperactivity disorder, Children, Children’s sleep habits questionnaire (CSHQ), EQ-5D-Y-3L, Health, Implementation, Melatonin, Sleep intervention, Weighted blanket

## Abstract

**Background:**

Sleep problems are common in children with attention-deficit/hyperactivity disorder (ADHD), yet the real-world use, implementation, and long-term outcomes of weighted blankets and standard melatonin treatment remain understudied. This study protocol describes a research project evaluating the real-world effectiveness and implementation of a weighted blanket intervention and standard melatonin treatment for children with ADHD and sleep problems in routine clinical practice. The project focuses on (1) changes over time in sleep, health-related outcomes, and resource utilization within and across treatment arms, and (2) barriers and facilitators to implementation in routine clinical practice.

**Methods:**

The research project uses: (1) a pragmatic longitudinal cohort design to investigate real-world changes over time in sleep, health-related outcomes, and healthcare resource utilization among children receiving either a weighted blanket intervention or standard melatonin treatment, and (2) a process evaluation design to evaluate implementation in routine clinical practice. Approximately 150 child-parent dyads will be included in each treatment arm. Children aged 6–14 years and one of their parents will each complete questionnaires at baseline, before treatment starts, at 1, 3, 6, and 12 months, and then annually for an additional nine years. The questionnaires assess sleep problems using the Children’s Sleep Habits Questionnaire (CSHQ) total score as the primary sleep outcome, treatment adherence and treatment changes, additional sleep-related measures, everyday function, lifestyle habits, worry and anxiety, ADHD-related symptoms, health-related quality of life, pain, resource utilization, and treatment use and implementation. Child-parent dyads and clinical staff members will be interviewed at 6 and 12 months. Appropriate parametric and non-parametric statistical tests will be performed. Interviews will be analyzed using qualitative content analysis. The implementation process will be analyzed using a convergent parallel mixed methods approach.

**Discussion:**

The project has the potential to inform clinical guidelines and decision-making frameworks for personalized care for children with ADHD. By evaluating patterns of treatment use and treatment change, real-world outcomes, and implementation in routine clinical practice, the project may contribute knowledge about the role of weighted blankets as a non-pharmacological treatment option alongside standard melatonin treatment.

**Trial registration:**

http://ClinicalTrials.gov, NCT06808425; registered 29 January 2025.

## Background

Attention-deficit/hyperactivity disorder (ADHD) is a neurodevelopmental condition that involves difficulties with concentration, hyperactivity and impulsivity [1–3]. It affects 6–8% of children [4] and is twice as common among boys as it is among girls (10% vs. 5%) [5]. ADHD negatively affects health-related quality of life [6], school performance [7], and family life [8], and entails significant costs to society [9]. Among children with ADHD, up to 73% experience sleep problems [10], compared to 23% of healthy peers [11], with a higher prevalence among girls than boys (75% vs. 53%) [12]. Sleep problems may be transient, but approximately 10% have persistent problems lasting more than one year [13]. Common sleep problems are bedtime resistance, difficulties falling asleep, nightly awakenings, difficulties with morning awakenings, reduced sleep efficiency, and reduced total sleep time [14, 15], which may lead to daytime sleepiness [16, 17] and negative impact on quality of life, social functioning [16], and ADHD symptoms [15]. Sleep problems in this population also impact negatively on parental mental health [18]. To alleviate ADHD symptoms and facilitate everyday life, children are often treated with stimulants, which can induce or exacerbate sleep problems, such as insomnia [2].

Sleep problems can be treated pharmacologically and/or non-pharmacologically. Prescription of the pharmacological treatment melatonin has increased significantly in Sweden in recent decades [19], and about one third of children with ADHD in Sweden are prescribed sleep medication [20]. Melatonin has demonstrated safety and effectiveness, but side effects such as headache, nausea and fatigue have been reported [21] and long-term effects are insufficiently described. Many parents prefer non-pharmacological treatments to pharmacological treatments [22]. Non-pharmacological treatments commonly include interventions targeted at sleep hygiene, behavioral therapy or sensory aids, such as weighted blankets [23]. Weighted blankets have long been used as a complement to or replacement for pharmacological treatment for children with ADHD and sleep problems [24]. Although widely used in clinical practice, the effects of sleep interventions with weighted blankets have been sparsely studied [25].

In 2020, because of a lack of evidence of their effectiveness, the Medical Technology Product Council in Sweden recommended that weighted blankets for sleep problems should only be prescribed in research studies. This recommendation was then included in the updated treatment guidelines published by the Swedish National Board of Health and Welfare in 2024 [26]. During the same period, our research group conducted a randomized controlled trial in which weighted blankets were compared with lighter control blankets in children with ADHD and sleep problems [27, 28]. Sleep was evaluated both objectively and subjectively by sleep actigraphy, questionnaires, and interviews [27, 29, 30]. Weighted blankets were found to increase total sleep time (mean difference 7.72 min) and sleep efficiency (mean difference 0.82%) and reduce nightly awakenings (mean difference − 2.79 min) and perceived sleep problems compared with the lighter control blankets. They were particularly effective for children 11–14 years of age with the inattentive subtype of ADHD. Results from sleep actigraphy did not support any changes in sleep onset [27]. The children experienced that the weighted blanket required adjustment to individual needs and preferences and adaptation to the environment, improved their ability to regulate worry and anxiety, changed sleep patterns, and promoted functioning in everyday life [29]. Parents experienced improved sleep routines, faster sleep onset, fewer awakenings, and increased well-being in their children, which in turn improved the children’s ability to manage school, leisure activities, and family life [30].

The studies by our research group have established knowledge about the development, feasibility, and evaluation of a weighted blanket sleep intervention for children with ADHD and sleep problems. The final phase, implementation, focuses on how the intervention works in clinical practice [31] to ensure that it is feasible, (cost-)effective, transferable, and scalable. The aim of this study protocol is to describe a research project evaluating the real-world effectiveness and implementation of a weighted blanket intervention and standard melatonin treatment in routine clinical practice for children with ADHD and sleep problems. The project focuses on: (1) effectiveness, including changes in sleep-related outcomes, treatment adherence, health-related outcomes, and resource utilization within and across the initial treatment arms during the first 12 months after inclusion, (2) longer-term patterns of treatment use, adherence, treatment changes, sleep, health-related outcomes, and resource utilization during the annual follow-ups, and (3) barriers and facilitators in implementation in routine clinical practice. We hypothesize that the two initial treatment arms will show different patterns of change in sleep-related outcomes during the first 12 months, but that both groups will improve according to the CSHQ total score. Changes in the standard melatonin treatment arm are expected to primarily concern sleep onset, whereas changes in the weighted blanket intervention arm are expected to concern other aspects of sleep, including total sleep time, nightly awakenings, and perceived sleep quality. We further hypothesize that approximately half of the children starting with a weighted blanket will continue using it at the one-year follow-up. The annual follow-ups will be used to describe longer-term patterns of continued use, discontinuation, switching between treatments, and combined use of weighted blankets and melatonin.

## Methods

### Study design

The research project uses a pragmatic prospective longitudinal cohort design with two initial treatment arms: a weighted blanket intervention and standard melatonin treatment. Treatment selection will be based on family preference and clinical decision-making in routine clinical practice rather than randomization. Child-parent dyads will be followed from baseline to 12 months, with annual long-term follow-ups for an additional nine years. The rationale for the long-term follow-up is to capture children’s development from childhood through adolescence and into young adulthood. The main evaluation of real-world changes in sleep, treatment adherence, health-related outcomes and resource utilization will focus on the first 12 months after inclusion, whereas the annual long-term follow-ups will be exploratory and used to describe longer-term patterns of treatment use, adherence, treatment changes, sleep, health-related outcomes, and resource utilization. Treatment changes may include continued use, discontinuation, switching between treatments, and combined use of weighted blankets and melatonin. The cohort study is combined with a process evaluation and uses a hybrid type 1 effectiveness–implementation approach [32, 33], in which clinical outcomes are the primary focus and implementation outcomes and contextual determinants are explored in parallel. Quantitative, qualitative, and mixed methods will be used.

### Patient and public involvement

The research project has been planned in collaboration with the Child and Adolescent Mental Health Service (CAMHS) and its ADHD subunit in Region Halland in terms of recruitment (inclusion and exclusion criteria and process and workflow of recruitment), preparation of intervention, and selection of outcome variables. Moreover, because the project has an implementation focus, emphasis has been placed on finding solutions that are adapted to clinical practice and to do so in coproduction with clinical staff.

Apart from disseminating results through scientific peer-reviewed articles within the research community, the findings from the research project will be disseminated through popular science reports, clinical conferences, and communication, with a special focus on patient organizations, healthcare organizations, and national and regional policymakers.

### Participants and recruitment

Children aged 6–14 who are diagnosed with ADHD (according to the Diagnostic and Statistical Manual of Mental Disorders [DSM], Fifth Edition) [34], screen positive for sleep problems, and are patients at the ADHD unit at CAMHS in the south-west of Sweden are eligible for inclusion in the research project. Children with presumed uncomplicated ADHD are triaged to the ADHD unit, whereas children with significant comorbidity or complex psychosocial needs requiring more intensive care are referred to regular CAMHS outpatient care and are not eligible for inclusion [35]. Approximately 350 children with presumed uncomplicated ADHD are seen annually at the ADHD unit, and based on clinical estimates, approximately half of them, about 175 children per year, are expected to screen positive for sleep problems and therefore be potentially eligible for the study. Children, and one of their parents, will be asked to participate in the research project. At inclusion, participants will enter one of two initial treatment arms based on family preference and clinical decision-making: a weighted blanket intervention or standard treatment with melatonin. Recruitment of child-parent dyads is expected to continue until approximately 150 children have been enrolled in each of the weighted blanket intervention and standard melatonin treatment groups.

When families first seek treatment for their child at CAMHS, they have a short telephone interview as part of a structured triage. After triage, the children see a physician for assessment and care planning. If applicable, ADHD medication is also prescribed. At this visit, children and parents receive verbal and written information about the research project and are given a sleep problems screening form to fill out and bring to the follow-up visit.

The sleep problems screening form is completed by parents and consists of three items from the Swedish version of the Children’s Sleep Habits Questionnaire (CSHQ-SWE) [36, 37]. Each item is scored in terms of 0–1, 2–4, or 5–7 days per week. The items are: “Child falls asleep within 20 minutes after going to bed” (scoring criteria 0–1 or 2–4 days per week), “Child sleeps too little” (scoring criteria 2–4 or 5–7 days per week), and “Child awakes more than once during the night” (scoring criteria 2–4 or 5–7 days per week). Each item contains the follow-up question: “Is this a problem?” (yes / no). If at least one of the items fulfills the scoring criteria and is indicated to be a problem, the child is considered eligible for inclusion.

At the follow-up visit, the sleep problems screening form is assessed by a nurse. If the child fulfills the inclusion criteria of ADHD diagnosis, sleep problems, age 6–14 years, and is a patient at the ADHD unit, and if the child-parent dyad understands written and spoken Swedish, the child-parent dyad is invited to participate in the research project.

Recruitment to the research project will occur continuously and include both newly and previously diagnosed children at the unit, and those who use and do not use ADHD medication. Participants will enter one of two initial treatment arms: (1) weighted blanket or (2) standard treatment with melatonin. During follow-up, participants may continue their initial treatment, discontinue treatment, switch between weighted blanket and melatonin treatment, or use the two treatments in combination, based on family preference and clinical decision-making. Treatment use and treatment changes over time will be documented as part of the pragmatic evaluation.

Follow-up by healthcare professionals at the ADHD unit will be integrated into the standardized care pathway. At inclusion, they will provide information on available treatment options and support families in initiating the selected treatment. During follow-up, healthcare professionals will discuss treatment use, perceived effects, adherence, and possible difficulties. Decisions about treatment continuation, switching, or combined use of weighted blankets and melatonin will be made in dialogue between the family and healthcare professionals as part of routine clinical practice. The standardized care plan for this group is the following: Parents and adolescents aged 15 years or older are offered a one-day course about ADHD. During the first year, follow-up visits are scheduled four weeks after initiation of new medication, and after six months, regardless of medication use. After the first year, the minimum follow-up is a yearly visit up to age 18, with an additional visit if a new medication is introduced. Any anxiety or depressive episodes will be referred to primary psychiatric care up to age 18 years. More complicated psychiatric issues will be referred to the specialized child and adolescent psychiatric services within the same provider. After age 18, the care will be referred to adult psychiatry. If medication is discontinued for more than one year, regular contact with the unit will end, but care can be reinitiated if needed.

### Intervention

The weighted blankets are fibre blankets (Novista.se) which distribute the weight evenly across the body, in comparison with other weighted blankets, such as ball blankets [38] or chain blankets [39], where the pressure is concentrated on certain points on the body [38, 39]. Children included in the research project receive a weighted blanket (4 kg, 6 kg, 8 kg, or 10 kg) with weight based on age, body weight, and degree of sleep problems, according to a specifically designed manual that follows practice for prescribing blankets. Melatonin will start at 1 or 2 mg, with a dose range up to 6 mg.

### Data collection and assessment

The child-parent dyads answer individual questionnaires at baseline (before the intervention starts), at 1-, 3-, and 6-month follow-ups, at 1-year follow-up, and then annually for an additional nine years. Parent-reported measures include both parent self-reports and parent proxy-reports concerning the child. The questionnaires contain questions about sleep problems, everyday function, lifestyle habits, worry and anxiety, ADHD-related symptoms, health-related quality of life, pain, resource utilization, and questions related to the intervention (Table 1). A purposive sample of approximately 20–25 child-parent dyads from each treatment arm will be interviewed individually at 6 and 12 months.

The implementation of the intervention will be evaluated by resource utilization and interviews. Approximately 5–10 individuals from the clinical staff at the ADHD unit will be interviewed individually and/or in focus groups at 6 and 12 months.

**Table 1:** Overview of outcome measures

		Child-reported measures	Parent-reported measures	Clinical staff–reported measures
**Sociodemographic variables**			Children: age, gender, country of birth, medical information, siblings, and age of siblings *. Parents: age, gender, country of birth, civil status, educational level, and employment. Partners: age, country of birth, educational level, and employment.	
**Primary outcomes**	Sleep problems		Children’s Sleep Habits Questionnaire (CSHQ) [36, 37] *, **	
	Treatment adherence and treatment changes		Usage of weighted blanket and/or melatonin *	
**Additional sleep-related measures**	Sleep problems	Insomnia Severity Index (ISI) [40] Sleep Self-Report (SSR) [41, 42]	Pediatric Insomnia Severity Index (PISI) [43] *	
**Secondary outcomes**	Everyday function	Child Outcome Rating Scale (CORS) [44] Work and Social Adjustment Scale, Youth version (WSAS-Y) [45]	Outcome Rating Scale (ORS) [46] Work and Social Adjustment Scale, parent version (WSAS-P) [45] * The Brief Child and Family Phone Interview (BCFPI) [47, 48] *	
	Lifestyle habits		Dietary habits, physical activity, screen time and sedentary time (Health Behaviour in School-aged Children (HBSC) survey) [49] *	
	Worry and anxiety	The State-Trait Anxiety Inventory – short form (Short STAI) [50–52]		
	ADHD-related symptoms		The Swanson, Nolan, and Pelham Scale (SNAP-IV parent) [53, 54] *	
	Health-related quality of life	EQ-5D-Y-3 L [55, 56]	EQ-5D-3 L [57, 58]	
	Pain	Visual Analogue Scale (VAS) [59]		
	Resource utilization		Treatment Inventory Cost in Psychiatric Patients (TIC-P) [59] School absence and healthcare consumption [60, 61] *	Healthcare consumption Costs of implementation
	Evaluation of intervention implementation			Checklist: Extent of implementation, accessibility and participant recruitment
	Interviews	Individual interviews regarding the sleep intervention: 1) Experiences of the interventions’ effects 2) Barriers and facilitators in the implementation process	Individual interviews regarding the sleep intervention: 1) Experiences of the interventions’ effects 2) Barriers and facilitators in the implementation process	Individual and/or focus group interviews on barriers and facilitators in the implementation of the sleep intervention

* Parent proxy-report concerning the child

** Primary sleep outcome for the main effectiveness evaluation

#### Sociodemographic variables

Parents will be asked to report their gender (man/woman/prefer not to say), child’s gender (boy/girl/prefer not to say), age, child’s age, civil status (single/spouse or partner), partner age, birth country, birth country of child, birth country of partner, highest educational level achieved (compulsory school/upper secondary school/university or college), partner’s highest educational level achieved, employment status (full time/part time/unemployment/parental leave/studies/sick leave or sickness benefit/other), partner’s employment status, number of siblings, and sibling(s’) age(s). Parents also report child comorbidity (no/yes and diagnoses), child medication (no/yes and type of medicines), child’s need for sleep medication (no/yes), and completion of treatment for sleep problems for the child during the last month (no/yes and type of treatment). An open-ended question enabling additional important information to be communicated is also included.

#### Primary outcomes

In relation to the main evaluation of real-world effectiveness, two main outcome areas will be studied: sleep problems and treatment adherence. The primary sleep outcome will be parent-reported sleep problems assessed by the Children’s Sleep Habits Questionnaire (CSHQ) total score. Treatment adherence and treatment changes over time will be assessed by studying continued use, discontinuation, switching between weighted blanket and melatonin treatment, combined treatment use, and withdrawal from the study.

##### Sleep problems

###### Children’s sleep habits questionnaire (CSHQ)

The Children’s Sleep Habits Questionnaire (CSHQ) [37], Swedish version (CSHQ-SWE) [36] is a parent-reported questionnaire assessing sleep during the past week in children by 33 questions. The frequency of sleep problems (questions 1–31) or sleepiness during various activities (questions 32–33) for each question is answered on a three-point scale (1–3 points). The questions are divided into eight dimensions: (1) Bedtime resistance, (2) Sleep onset delay, (3) Sleep duration, (4) Sleep anxiety, (5) Night wakings, (6) Parasomnias, (7) Sleep-disordered breathing, and (8) Daytime sleepiness. In addition to dimensions, the severity of sleep problems is summed into a total score (range 33–99), where a higher score indicates a greater degree of sleep problems [37]. The clinical cut-off for sleep problems in school-aged children with ADHD is 48 [40]. Non-scored inquiries encompass the child’s bedtime, the time the child fell asleep, the duration of night sleep and naps, the number and duration of night wakings, and the time of morning awakening [36]. The questionnaire was originally validated in 4–10 year-old children [37], but has been used in children up to 12 years of age [41]. The Swedish version is validated for use in children with ADHD aged 6–12 [36].

##### Treatment adherence

Treatment adherence and treatment changes over time will be assessed by studying continued use, discontinuation, switching between weighted blanket and melatonin treatment, combined treatment use, and withdrawal from the study.

#### Additional sleep-related measures

Additional sleep-related measures will be used to capture different aspects of sleep and to include both parent proxy-reported and child self-reported perspectives across the broad age range and follow-up period.

##### Sleep problems

###### Insomnia severity index (ISI)

The Insomnia Severity Index (ISI) [42] assesses insomnia in the past two weeks by seven questions. The first questions encompass difficulties with: (1) Sleep onset, (2) Sleep maintenance, and (3) Early morning awakening. The remaining questions concern: (4) Satisfaction with current sleep pattern, (5) Interference with daily functioning, (6) Noticeability of impairment attributed to the sleep problem, and (7) Level of distress caused by the sleep problem. A version will be used that has been adapted specifically for children. The children rate the severity of sleep problems on a five-point scale (0–4), which is summed to a total score (0–28), with higher scores indicating greater sleep problems [42].

###### Pediatric insomnia severity index (PISI)

The Pediatric Insomnia Severity Index (PISI) [43, 44] is a parent-reported questionnaire assessing insomnia during the past week in children by six questions, encompassing difficulties with: (1) Sleep onset delay, (2) Sleep onset, (3) Nightly awakenings, (4) Falling back to sleep after nightly awakenings, (5) Daytime sleepiness, and (6) Sleep duration. The parents rate the frequency of sleep problems on a six-point scale (0–5 points), and the severity of sleep problems is summed into a total score (range 0–30 points), where a higher score indicates a greater degree of sleep problems. PISI is validated in 4–10-year-olds [43, 44], but has also been used in parents with children aged 10–12 [45].

###### Sleep self-report (SSR)

The Sleep Self-Report (SSR) [46, 47] is a child-reported questionnaire that assesses frequency of sleep-related difficulties with a focus on bedtime, sleep behavior and daytime sleepiness. The SSR contains 26 questions, of which 23 are answered on a three-point scale (1–3 points), and the remaining three are non-scored inquiries. The total score ranges from 23 to 69, with a higher score indicating more sleep problems [46]. The questionnaire was originally validated in 7–12-year-olds [46], but has also been used up to the age of 18 [48, 49]. SSR shows sufficient sensitivity to distinguish a clinical population from a general population [50].

#### Secondary outcomes

##### Everyday function

###### Child outcome rating scale (CORS)

The Child Outcome Rating Scale (CORS) [51] assesses everyday function and well-being in the domains of individual, family, school, and overall. Each domain is rated by the child on visual analogue scales (VAS) ranging from 0 (frowning face) − 10 (happy face), where 10 corresponds to the best. In addition, a total score ranging from 0 to 40 (worst–best) will be utilized. CORS demonstrates satisfactory validity and reliability [51].

###### Outcome rating scale (ORS)

The Outcome Rating Scale (ORS) [52] assesses parents’ everyday function and well-being in the domains of individual, interpersonal, social role, and overall. Each domain is rated by the parent on VAS, ranging from 0 to 10, where 10 corresponds to the best. In addition, a total score ranging from 0 to 40 (worst–best) will be utilized. ORS demonstrates satisfactory validity and reliability [52].

###### Work and social adjustment scale, youth version (WSAS-Y)

The Work and Social Adjustment Scale, Youth version (WSAS-Y) [53] assesses children’s daily functioning. The instrument consists of five questions where children estimate on a nine-point scale (0–8 points) the extent to which their problems affect school, daily skills, social activities, hobbies, and family and relationships. A total score ranging from 0 to 40 is obtained, with higher scores indicating greater adverse impact. WSAS-Y demonstrates satisfactory validity and reliability [53]. A version specifically adapted to children with ADHD will be used.

###### Work and social adjustment scale, parent version (WSAS-P)

The Work and Social Adjustment Scale, Parent version (WSAS-P) [53] assesses children’s daily functioning. The instrument consists of five questions where parents estimate on a nine-point scale (0–8 points) the extent to which the children’s problems affect school, daily skills, social activities, hobbies, and family and relationships. A total score ranging from 0 to 40 is obtained, with higher scores indicating greater adverse impact. WSAS-P demonstrates satisfactory validity and reliability [53]. A version specifically adapted to parents assessing children with ADHD will be used.

###### The brief child and family phone interview (BCFPI)

The Brief Child and Family Phone Interview (BCFPI) [54, 55] is a screening tool consisting of scales for DSM-related symptoms that is used in CAMHS to triage mental health conditions in children. Selected scales will be utilized: “Impact on child functioning” (social participation, quality of social relations, and school participation and achievement), “Impact on family functioning” (family activities and family comfort), and “Parental functioning” (appetite problems, concentration problems, and emotional problems). The frequency of each event is rated by the parents on Likert scales (best–worst). BCFPI demonstrates satisfactory validity and reliability [54, 55].

##### Lifestyle habits

###### Lifestyle habits

The child’s lifestyle habits will be estimated by parents by selected questions included in the Health Behaviour in School-aged Children (HBSC) survey carried out by the Public Health Agency of Sweden [56]. The child’s height and weight, dietary habits (fruit intake, vegetable intake, consumption of sweets, and consumption of sugar-sweetened beverages), physical activity (fulfillment of recommendations, frequency of and time spent on exercise during leisure time) and screen time and sedentary (time spent in front of screen-based entertainment, time spent in sedentary gaming, and time spent using electronic devices for other purposes) are assessed [56].

##### Worry and anxiety

###### The state-trait anxiety inventory scale – short form (Short STAI)

The State-Trait Anxiety Inventory Scale – short form (Short STAI) [57, 58, 62] assesses anxiety in children by six questions encompassing the themes of Calm, Tense, Upset, Relaxed, Content, and Worried. Each question is rated by the child on a four-point Likert scale (1–4 points), and the total score ranges from 6 to 24 points with higher scores indicating more anxiety [57, 58]. Short STAI demonstrates satisfactory validity and reliability [57, 58].

##### ADHD-related symptoms

###### The swanson, nolan, and pelham scale – parent-reported version (SNAP-IV parent)

The Swanson, Nolan, and Pelham Scale – parent-reported version (SNAP-IV parent) [59, 60] assesses children’s ADHD symptoms and Oppositional Defiant Disorder (ODD) symptoms. The questionnaire includes 30 questions rated by the parent on a four-point Likert scale (0–3 points). Attention deficit is covered by nine items and scored from 0 to 27 points. Hyperactivity and impulsivity are combined, covered by six items and three items respectively, and scored from 0 to 27 points. ODD is covered by eight items and scored from 0 to 24 points. A higher score reflects greater severity of symptoms. The additional four items are supplementary to ADHD (two items) and ODD (two items) and will not be included in scoring [59, 60]. SNAP-IV parent demonstrates satisfactory validity and reliability [59, 61].

##### Health-related quality of life

###### EQ-5D-Y-3L

The EQ-5D-Y-3L [63, 64] is a child-reported measure that assesses health-related quality of life in children. The questionnaire covers five domains: mobility, looking after myself, doing usual activities, having pain or discomfort, and feeling worried, sad or unhappy. Problems within each domain are rated by the child on a three-point Likert scale (1–3 points) and re-calculated into an index ranging from 0.00 to 1.00 (worst–best). The questionnaire also includes a VAS (0–100 points, worst–best) to reflect current health state [63, 64]. EQ-5D-Y-3L demonstrates satisfactory validity and reliability [64].

###### EQ-5D-3L

The EQ-5D-3L [65–67] assesses health-related quality of life in adults. The questionnaire covers five domains: mobility, self-care, usual activities, pain/discomfort, and anxiety/depression. Each domain is rated by the parent on a three-point Likert scale (1–3 points) and re-calculated into an index ranging from 0.00 to 1.00 (worst–best). The questionnaire also includes a VAS (0.00–100.00 points, worst–best), reflecting current health state [65–67]. EQ-5D-3L demonstrates satisfactory validity and reliability [67–69].

##### Pain

###### Pain

Pain intensity during the last week is assessed by a VAS. The child estimates their perceived pain intensity on a scale from 0 to 100, where 0 corresponds to “no pain” and 100 corresponds to “worst pain possible”. VAS has been recommended for assessment of chronic pain in children [70].

##### Resource utilization

###### Treatment inventory cost in psychiatric patients (TIC-P)

The Treatment Inventory Cost in Psychiatric Patients (TIC-P) [71] is a questionnaire that assesses absence from work and changes in work productivity for parents. Six selected items covering those aspects will be utilized. TIC-P demonstrates satisfactory validity and reliability [71].

###### School absence and healthcare consumption

Absence from school and healthcare consumption for the children, such as number of visits to medical doctors, nurses, psychologists, emergency departments and specialist care providers, will be reported by parents. The questions have been used in previous studies [72, 73]. Healthcare consumption also includes clinical registry data from the medical health records of the participating children, including the number of visits to the CAMHS and the ADHD unit, type of visit, healthcare professional involved, and prescribed pharmacological treatment.

###### Costs of implementation

Costs of implementation include the costs of prescription of weighted blankets or melatonin, the healthcare region’s purchase price of weighted blankets or melatonin, administrative costs, and clinical staff time.

##### Evaluation of intervention implementation

###### Usage of weighted blanket and/or melatonin

Parents will report frequency of usage of weighted blanket and/or melatonin on a 4-point scale (every night = 6–7 nights a week; almost every night = 4–5 nights a week; sometimes = 2–3 nights a week; almost never = 0–1 night a week). An additional three open-ended questions will be available concerning how weighted blankets and/or melatonin have been used, the reason for usage, and thoughts about usage.

###### Checklist

A checklist will be maintained by clinical staff and researchers in the project to evaluate the extent of implementation, accessibility and recruitment of participants, and treatment use over time, including continued use, discontinuation, switching between treatments, combined use of weighted blankets and melatonin, and withdrawal from the study.

##### Interviews

###### Interviews with children and parents

Individual interviews will address the effects of the intervention (weighted blanket or standard treatment with melatonin) and how sleep affects the child’s everyday functioning, everyday activities, worry and anxiety, ADHD-related symptom burden, health-related quality of life, sensory impressions, and parents’ health, and will include questions about the implementation of the intervention and participation in the research project. Probing questions will be used to elicit in-depth responses.

###### Interviews with clinical staff

Individual interviews and/or focus group interviews will be carried out at the ADHD unit with multi-disciplinary clinical staff, addressing the barriers and facilitating factors in the process of implementing the sleep intervention in clinical practice, resources, and strategies utilized. Probing questions will be used to elicit in-depth responses.

### Statistical power

The primary outcome is the CSHQ total score. In the absence of an established minimal clinically important difference for the CSHQ and robust estimates of the expected effect of melatonin on CSHQ scores, the sample size calculation was based on previous publications from the SLEEP project, including the randomized controlled crossover trial and subsequent analyses of the same cohort [27, 74, 75], together with studies of melatonin interventions using the CSHQ [76, 77]. In the SLEEP project, the baseline mean CSHQ total score was approximately 53, with an SD of approximately 8, and an assumed follow-up mean of 47 was used for the weighted blanket intervention. Studies of melatonin interventions have reported improvements of approximately 10 points, corresponding to an assumed follow-up mean of 43, with an SD of 8 [76, 77]. Based on the assumed follow-up means of 47 and 43 and a common SD of 8, the standardized effect size was estimated as Cohen’s d = (47 − 43)/8 = 0.50. With 80% statistical power and a two-sided alpha of 0.05, 64 participants are required per treatment group. Given the uncertainty in the assumptions underlying the sample size calculation, and to allow for expected attrition, switching between treatments, combined use of weighted blankets and melatonin during the first 12 months, and retention of a sufficient sample for longer-term follow-up analyses, we aim to include 150 children per initial treatment arm, for a total of 300 children.

### Data analysis

#### Quantitative methods

Data analysis will be performed using IBM SPSS Statistics software v.28 or later (IBM Corp., Armonk, NY, USA). Continuous data will be presented as means and standard deviations or medians and interquartile ranges, and categorical data as numbers and percentages. Changes over time in continuous outcomes assessed repeatedly, including the primary sleep outcome, additional sleep-related measures, and health-related outcomes, will be analyzed using repeated-measures analysis of variance (R-ANOVA) or repeated-measures analysis of covariance (R-ANCOVA), as appropriate. Analyses to assess real-world effectiveness within and across the initial treatment arms will include the primary sleep outcome, treatment adherence, health-related outcomes, and resource utilization, using appropriate statistical methods, such as t-tests, chi-squared tests, or analysis of covariance (ANCOVA). When appropriate, age, sex, ADHD medication, and level of ADHD symptoms, will be included as covariates in R-ANCOVA or ANCOVA models. Analyses of long-term follow-up will take into account patterns of treatment use and changes over time, including continued use, discontinuation, switching between treatments, and combined use of weighted blankets and melatonin. Differences in resource utilization will be analyzed through non-parametric analyses of individual data. To address the risk of Type I errors due to multiple comparisons across several outcome measures, Bonferroni corrections will be applied where appropriate.

#### Qualitative methods

Interviews with children and parents will be analyzed using inductive qualitative content analysis. Interviews will be audio-recorded and transcribed verbatim. Meaning units adhering to the aim will be extracted, condensed, and coded. The codes will be categorized according to similarities and differences and will be further grouped into subcategories and categories. Both manifest and latent content may be analyzed [78, 79].

#### Mixed methods

The implementation process will be analyzed with a convergent parallel mixed method [80] that includes both descriptive statistics and qualitative content analysis of implementation outcomes, barriers, and facilitators in routine clinical practice, and treatment adherence, patterns of treatment use, and treatment changes over time. This includes continued use, discontinuation, switching between treatments, and combined use of a weighted blanket and melatonin from the perspectives of clinical staff, children and parents. Changes in clinical routines, staffing, and implementation procedures over time will be documented and considered as contextual factors in the process evaluation and interpretation of the long-term findings.

The implementation evaluation will combine an implementation outcomes framework, a process evaluation framework, and a determinants framework. Proctor’s Implementation Outcomes Framework [81] will be used to conceptualize relevant implementation outcomes, including acceptability, feasibility, fidelity, reach, implementation cost, and sustainability, where applicable. Moore et al.’s process evaluation framework [82] will be used to structure process-related components of the evaluation, including fidelity, dose, reach, recruitment, exposure, adherence, and satisfaction. Contextual determinants, including barriers and facilitators, will be analyzed using the Consolidated Framework for Implementation Research (CFIR) as a determinants framework [83] (Table 2).

1. Intervention: Characteristics of the intervention, e.g., perceived adaptability and complexity for use in routine practice.
2. External environment: Characteristics of the external environment, e.g., patient needs, resources, external policies and incentives.
3. Internal environment: Characteristics of the environment in which the intervention is implemented, e.g., structural characteristics and culture.
4. Individual domain: Characteristics of the clinical staff members delivering the intervention, e.g., knowledge, attitudes and confidence regarding the intervention being implemented.
5. Implementation process: The activities and strategies used to support the implementation of the intervention, e.g., planning and execution.

**Table 2:** Evaluation of implementation outcomes, process components, and contextual determinants

	Questions about the evaluation of the intervention	Data collection	Data analysis
1. Fidelity	To what extent was the intervention implemented as planned?	Interviews with clinical staff.	Descriptive analysis.
2. Dose	To what extent was the weighted blanket intervention delivered in routine clinical practice?	Clinical staff and researcher checklist (documentation of sleep problems in children and the number of those who were offered and agreed to participate in the research project).	Descriptive analysis.
3. Reach	To what extent were eligible children and families reached and included in the research project?	Clinical staff and researcher checklist and follow-up every 3 months. Individual interviews and/or focus groups with clinical staff.	Descriptive analysis.
4. Recruitment	How were children and families recruited to the sleep intervention?	Clinical staff and researcher checklist and follow-up every 3 months. Individual interviews and/or focus groups with clinical staff.	Descriptive analysis.
5. Exposure, adherence, and satisfaction	To what extent did children continue, discontinue, switch, or combine treatments during follow-up? Did children, parents, and clinical staff find the sleep intervention acceptable and satisfactory?	Clinical staff and researcher checklist. Parent-reported use of weighted blanket and/or melatonin. Individual interviews and/or focus groups with clinical staff, parents and children.	Descriptive analysis. Categories identified through content analysis.
6. Implementation cost	What costs and resources were associated with implementing the weighted blanket intervention and standard melatonin treatment?	Data on costs of prescription of weighted blankets or melatonin, purchase prices, administrative costs, and clinical staff time	Descriptive analysis.
7. Context	What barriers and facilitators influenced implementation of the sleep intervention? The five CFIR domains concern characteristics of: 1. Intervention 2. External environment 3. Internal environment 4. Individuals 5. Implementation process	Individual interviews and/or focus groups with clinical staff, parents and children at 6 and 12 months.	Categories identified through content analysis.

Implementation outcomes are informed by Proctor’s Implementation Outcomes Framework [81]; process-related components are structured according to Moore et al.’s process evaluation framework [82]; and contextual determinants, including barriers and facilitators, are based on the Consolidated Framework for Implementation Research (CFIR) [83]

## Discussion

Weighted blankets have demonstrated potential to reduce perceived sleep problems [27, 29, 30]; however, to the best of our knowledge, no study has previously explored the process of implementing a weighted blanket intervention in clinical practice for children with ADHD and sleep problems. There is also a knowledge gap concerning real-world effectiveness, longer-term patterns of treatment use, adherence, and treatment changes, and implementation of a weighted blanket intervention and standard melatonin treatment in routine clinical practice. This research project will address these gaps by following sleep-related outcomes, treatment adherence, health-related outcomes, resource utilization, and treatment changes, including continued use, discontinuation, switching between treatments, and combined use of weighted blankets and melatonin, within and across the initial treatment arms over time. The results will inform future evaluation of treatment options for children with ADHD and sleep problems and support the implementation of weighted blanket interventions in clinical practice. Moreover, the assessment of various aspects of the health of children and their parents will add to knowledge on how the child’s ADHD diagnosis combined with sleep problems affects the family in their everyday lives.

Methodological considerations need to be discussed. A strength of the research project is that it includes both questionnaires and interviews. The questionnaires capture diverse and broad outcomes and will be answered by both children and parents. Moreover, the multi-informant design of interviews ensures that children’s, parents’ and clinicians’ perspectives are taken into consideration, strengthening the generalizability and applicability in clinical settings and everyday life. The use of quantitative, qualitative, and mixed methods ensures rich data, which could support validity of results.

A methodological challenge is to ensure participation in the longitudinal follow-ups. The battery of questionnaires is extensive, and follow-ups are frequent during the first year, which may reduce participants’ willingness to continue participating. To address this, each parent will be contacted by the research team at recruitment by phone so questions can be answered, aims revisited, and the importance of adherence can be emphasized.

A limitation of this research project is that some outcome measures will be administered outside their validated age ranges. This pragmatic approach reflects the broad age range of eligible participants, the continuous recruitment, and the relatively long follow-up period of the study cohort, allowing the use of a consistent assessment battery throughout the study. The selected instruments assess constructs that are considered theoretically relevant across the included age span. However, as their psychometric properties have not been established for all ages included, findings from participants outside the validated age ranges should be interpreted with caution and considered exploratory. Although the CSHQ total score has been specified as the primary sleep outcome for the main effectiveness evaluation, additional sleep-related measures are included to capture different aspects of sleep across the broad age range and follow-up period. As children grow older, more emphasis will be placed on self-reported instruments rather than proxy-reported instruments. However, the use of both proxy-reported and self-reported sleep measures throughout the study period allows overlap between perspectives and may strengthen comparisons over time. Another possible limitation is that sensory profiles will not be assessed by a specific measure. As the study incorporates multiple questionnaires, compromises are necessary to balance data collection needs with participant welfare. In consideration of the sensitive nature of the study population, the aim is to minimize respondent burden and avoid imposing excessively lengthy assessment protocols. Participants will be asked about their sensory impressions in interviews, which will enable in-depth information that may increase understanding of the sensory aspects of weighted blanket use for children with ADHD.

The research project, rooted in clinical practice, aims to inform clinical guidelines and decision-making frameworks for personalized ADHD care. Grounded in the real-world clinical setting, it is highly relevant to healthcare systems. By exploring the implementation of weighted blankets as a non-pharmacological treatment, the project may promote a multimodal approach to management of sleep problems in children with ADHD. This approach may provide families and healthcare professionals with a non-pharmacological treatment option alongside standard melatonin treatment.

## Data Availability

No datasets were generated or analysed during the current study.

## Abbreviations

ADHD: Attention-deficit/hyperactivity disorder
ANCOVA: Analysis of Covariance
BCFPI: Brief Child and Family Phone Interview
CAMHS: Child and Adolescent Mental Health Service
CFIR: Consolidated Framework for Implementation Research
CORS: Child Outcome Rating Scale
CSHQ: Children’s Sleep Habits Questionnaire
CSHQ-SWE: Children’s Sleep Habits Questionnaire – Swedish version
DSM: Diagnostic and Statistical Manual of Mental Disorders
HBSC: Health Behaviour in School-aged Children
ISI: Insomnia Severity Index
ORS: Outcome Rating Scale
ODD: Oppositional Defiant Disorder
PISI: Pediatric Insomnia Severity Index
R-ANCOVA: Repeated Measures Analysis of Covariance
R-ANOVA: Repeated Measures Analysis of Variance
SNAP-IV: Swanson, Nolan, and Pelham Scale IV
SSR: Sleep Self-Report
STAI: State-Trait Anxiety Inventory Scale
TIC-P: Treatment Inventory Cost in Psychiatric Patients
VAS: Visual analogue scale
WSAS-P: Work and Social Adjustment Scale, Parent version
WSAS-Y: Work and Social Adjustment Scale, Youth version
